# Descriptive Study of Attitudes towards Corporal Expression in Physical Education Students in a Region of Spain

**DOI:** 10.3390/healthcare11040549

**Published:** 2023-02-13

**Authors:** Jorge Rojo-Ramos, Noelia Mayordomo-Pinilla, María Mendoza-Muñoz, José C. Adsuar, David Manuel Mendoza-Muñoz, Carmen Galán-Arroyo

**Affiliations:** 1 Physical Activity for Education, Performance and Health (PAEPH) Research Group, Faculty of Sports Sciences, University of Extremadura, 10003 Caceres, Spain; 2 Promoting a Healthy Society Research Group (PHeSO), Faculty of Sport Sciences, University of Extremadura, 10003 Caceres, Spain; 3 Research Group on Physical and Health Literacy and Health-Related Quality of Life (PHYQOL), Faculty of Sport Sciences, University of Extremadura, 10003 Caceres, Spain; 4 Departamento de Desporto e Saúde, Escola de Saúde e Desenvolvimento Humano, Universidade de Évora, 7004-516 Evora, Portugal

**Keywords:** corporal expression, attitudes, physical education

## Abstract

(1) Background: In recent years, there has been increasing interest in understanding the factors that determine students’ attitudes and interest in learning. The information that can be extracted from students’ attitudes is essential for teachers to plan their classes to capture their attention and promote learning. Thus, this study aimed to determine whether there were significant differences between the genders in the perception of students from Extremadura towards Corporal Expression (CE) in Physical Education (PE) classrooms. (2) Methods: A single-measure descriptive and correlational cross-sectional study was conducted. A total of 889 PE students in the Compulsory Secondary Education (CSE) stage from public schools in Extremadura, Spain, participated in the study; the subject had a mean age of 14.58 (SD = 1.47) and a BMI of 20.63 (SD = 3.46). Variables related to gender, age, height, and weight of the participants and a questionnaire on attitudes towards Corporal Expression were included. (3) Conclusions: Girls showed a more positive perception of the CE contents of the PE subject than boys; the latter showed a greater indifference to and a lower preference for these contents compared to other contents of the subject. On a general level, participants valued CE with a certain degree of positivity regarding its formative and educational usefulness and the expression of feelings and emotional self-management, and the pupils agreed with the methods and means used by the teacher to transmit the learning of CE.

## 1. Introduction

The interest in knowing the factors that determine the attitudes and interests of students in learning has increased in recent years. The information that can be extracted from the attitudes of students is essential to the approach towards the classes on the part of teachers in order to capture their students’ attention and promote learning [1]. One of the didactic tools for the development of learning is Corporal Expression (CE) [2], a resource that enhances learner autonomy and competence as well as progress in learner relations [3]. Over time, attempts have been made to define CE in many different ways. In 1967, Stokoe defined it as a tool for communicating moods to reflect feelings so that people can get in touch with themselves and with others. Years later, it evolved into the development of the manifestation of the corporal as it did through its language, which consists of gestures, facial expressions, and postures [4]. Although the concept of CE varies from author to author according to the cultural factors surrounding it, there are specific elements that are repeated in each of these definitions. These elements are based on communication, creativity, and language combined with four opposing factors: intentionality, consciousness, spontaneity, and technique. In short, CE is a set of techniques and creative dimensions that an individual can develop through physical work to achieve the ability to express their emotions to others through the movement of their body and interaction with the environment using a language of their own that is composed of gestures, postures, and expressions that are characteristic of each individual and are capable of safeguarding the deficiencies of communication [5,6]. Among the characteristics developed via CE are creativity and those of multiple dimensions such as intellectual, physical, and sensory through techniques that enhance the capacity for attention and concentration for the integral development of students [1].

In the Spanish educational system, primary education (between 6 and 12 years of age), which is compulsory and free of charge, is divided into three cycles of two academic years each; Physical Education (PE) corresponds to a total of 100 h per cycle (50 h per year). At this stage, out of the four existing blocks of basic knowledge, the content of CE is specifically addressed through block D (“Music and performing arts”) in the three cycles, and the complexity of the content evolves progressively with the passing of the cycles [7]. Secondary education consists of a compulsory and free part (Compulsory Secondary Education (CSE)) comprising four courses divided into two two-year cycles (between the ages of 12 and 16). The PE subject corresponds to a total of 105 h for the first three years and 35 h for the fourth year. Of the six existing basic knowledge blocks, the content of CE at this stage is specifically addressed through block E (“Manifestations of Motor culture”), in which the contents of the block present a greater complexity at the physical, cognitive, and social levels in the second cycle [8].

CE was not part of the curriculum marked by the authorities in the contents to be taught until the inclusion of CE in the field of PE. This occurred due to the Organic Law 14/1970 of 6 August 1970 (General Law of Education and Financing of Education Law), in which it appears as Dynamic Expression (General Law of Education). This law evolved until the Organic Law 2/2006 of 3 May 2006 on education (LOE), in which CE was consolidated in PE and modified in 2020 to become LOMLOE [9]. In the community of Extremadura, these contents are part of the subject of PE throughout the first and second years of Compulsory Secondary Education (CSE) as well as in the third and fourth years of CSE; they advance from one cycle to another in the specificity of the techniques of expression and interpretation (DECRETO 110/2022, 2022) [10].

Factors that play a key role in CE are the attitudes of both teachers and students. Attitude and predisposition to the practice of these contents are largely determined by previous experiences in the subject; in the scientific literature, we found works in which most of the students had never had previous experiences with CE [11]. Moreover, CE-related activities were not among the students’ interests [12,13,14]. Teachers, on the other hand, had received little or no training on CE in their own studies, and in addition, before their training they had no experience related to CE due to its late inclusion in the educational curriculum [13,15]. The training of teachers and their previous experiences have a great influence on the approach of the teaching units towards CE, which in general is one of the contents that is less used in the classroom, resulting in a greater importance of and a better predisposition toward collective sports and games [1,2,15]. The wrong approach can condition students’ experiences and attitudes [14]. Another factor that conditions the attitudes of students is the perception experienced in the classroom, which is often limited by the teacher’s consideration of CE to be of little relevance in their preparation [1]. Furthermore, studies revealed that the teachers’ preferences were highly relevant in the creation of student preferences towards certain content and were differentiated among genders [16].

The research focused on attitudes on the part of students in CE based on gender differences. The practice of sports has been conditioned by gender stereotypes since its beginnings and continues to this day on an international level [17,18,19,20]. Studies report that in different geographical locations, the predisposition of girls towards PE is more negative than that of boys [20,21] As for PE content, boys preferred competitive activities and group sports and had a negative conception of CE, which caused them to develop negative feelings such as shame and weakness and to experience mockery, while girls had a better predisposition towards CE-related content [1,22,23]. Other research showed that these gender stereotypes are becoming less prominent and that the lines between the attitudes of the two genders are becoming increasingly blurred [1,24].

For these reasons, this study aimed to explore the perceptions of CE by students in PE classes in Extremadura as well as differences based on gender. Thus, we hypothesised that students’ perceptions of CE in PE classes would differ according to gender.

## 2. Materials and Methods

### 2.1. Study Design

A single-measure descriptive and correlational cross-sectional study was conducted.

### 2.2. Participants

A total of 889 students in PE at the CSE stage from public schools in Extremadura participated in the study. Of the total sample, 47.6% (n = 423) were boys and 52.4% were girls (n = 466). The mean age of the participants was 14.58 (SD = 1.47), and the mean BMI was 20.63 (SD = 3.46). A convenience sampling method was used to select the sample [25].

Table 1 shows the sociodemographic characterization of the sample.

### 2.3. Procedure

A convenience sampling method was used to access the sample [25]. First, the contact details of all the compulsory secondary schools in Extremadura (Spain) belonging to the Regional Ministry of Education and Employment were selected from the directory of public schools in Extremadura (Spain).

Secondly, an e-mail was sent to the PE teachers of all the schools that taught CSE to inform them of the purpose of the study. In addition, they were informed that if they wished to collaborate in the study, they should send the informed consent attached to the e-mail to all the families of the students in PE and that once the centre had collected all of the informed consents, they should make an appointment with the research team at the e-mail address provided.

Data collection was carried out between October 2021 and May 2022. The research team visited all of the educational centres that met the requirements of wishing to collaborate in the study and had the informed parental consents of the group of students to whom the questionnaire was to be administered. Before the completion of the forms in digital format using tablets owned by the research group, the research team read all the items of the instrument aloud to ensure that they were correctly understood and answered all the questions that were asked regarding the interpretation of the instrument. All data were collected and processed anonymously, and the average time to answer the form was 8 min.

### 2.4. Instruments

Sociodemographic questionnaire: A questionnaire was included with four questions regarding the gender, age, height, and weight of the participants.

Questionnaire of attitudes towards Corporal Expression: The questionnaire was used to measure attitudes towards CE [14] structured in four factors and composed of 32 items. Dimension 1 (“Valuation of Corporal Expression”) was composed of 14 items and evaluated the valuation and usefulness of CE on the part of the secondary education students. Dimension 2 (“Preference”) was composed of seven items that evaluated the value given to CE as opposed to other contents in the area of PE. Dimension 3 (“Pleasure or liking”) was composed of six items designed to evaluate positive attitudes that students showed towards CE. Finally, dimension 4 (“Attitude of the teacher”) was composed of five items that evaluated the perceptions that the students had regarding the actions of the teacher in CE classes. The responses used a five-point Likert scale with 1 being “strongly disagree” and 5 “strongly agree”. We determined the reliability of the questionnaire using the Cronbach’s alpha value and obtained a value of 0.94.

### 2.5. Statistical Analysis

All data were treated anonymously using SPSS statistical software version 23 for Mac (IBM SPSS, Chicago, IL, USA). To determine the choice of statistical tests to be used, the assumption of normality was explored using the Kolmogorov–Smirnov test, which reported that this assumption was not met. Therefore, nonparametric statistical tests were used.

To analyze the existence of statistically significant differences according to gender for each of the items and each of the dimensions of the questionnaire, the Mann–Whitney U test was used. To analyze the relationship between each of the dimensions of the instrument and the variables of age and body mass index (BMI), Spearman’s Rho test was used. Finally, Cronbach’s alpha was used to obtain the reliability values for each of the dimensions. The data are presented as the mean and standard deviation; the Bonferroni correction was applied for multiple comparisons, so that a significance value was established for each of the items (*p* < 0.0016) and each of the dimensions (*p* < 0.016).

## 3. Results

Table 2 shows the descriptive data and differences for each of the questionnaire items according to gender. Girls showed more agreement than boys with the 32 items of the questionnaire by scoring higher in all of them, showing statistically significant differences (*p* < 0.0016) in most of them, and showing a more positive attitude towards CE. 

Table 3 shows the descriptive data for each dimension according to gender and age group. Girls obtained higher scores than boys in all dimensions, and statistically significant differences were found in all of them. Therefore, girls rated the usefulness of CE more positively (1. Assessment of CE; *p* < 0.001), showed a greater preference for other contents of CE (2. Preference; *p* < 0.001), showed a greater interest in CE (3. Pleasure or liking; *p* < 0.001), and rated more positively the teacher’s attitude towards CE than boys (4. Teacher’s attitude *p* = 0.001). In addition, no significant differences were obtained in relation to the age groups.

In addition, reliability values were calculated for each of the four dimensions of the questionnaire by applying Cronbach’s alpha. Satisfactory values were obtained for all of them according to Nunally and Berstein [26]: α1 = 0.91, α2 = 0.71, α3 = 0.87, and α4 = 0.85.

Finally, the relationship between the different dimensions of the instrument for measuring attitudes towards CE and the variables of age and BMI was analyzed (Table 4). No significant associations were found between either variable.

## 4. Discussion

In the present research, the perceptions of CE among secondary school students in Extremadura were analyzed according to gender through a questionnaire composed of 32 items.

Among the main findings, statistically significant differences were found between the scores obtained in most of the items according to gender. In these items, the scores obtained by girls were higher than those obtained by boys; girls showed more agreement with the different items of this questionnaire on the perception of CE. Different investigations showed how the degree of approval and the interest of the students in a subject depended on the perception of feeling competent in the execution of the tasks demanded and the generation of gender stereotypes regarding the contents of the subject of PE [3,27]. Concerning this, several authors mentioned some existing views regarding CE content. These were subjected to stereotyped messages that associated CE with “dancing” and suggested that dancing was for girls and that boys should not show a preference for dancing because it can be understood as a weakness, thereby implying a greater motivation regarding this content on the part of girls [17,28]. In contrast to this thinking, in a study by Bonet and Menescardi Royuela, the results showed that most of the students thought that gender did not influence attitudes towards CE contents; that is, students did not show negative feelings or inappropriate behaviours (teasing, laughter, etc.) when boys practiced CE activities [1].

Regarding the four dimensions established by the questionnaire, statistically significant differences were found between the genders in all dimensions, and girls obtained higher scores in all four dimensions. In the dimension “Valuing Corporal Expression”, the students showed some agreement (M = 3.70) with the items of the questionnaire that corresponded to this dimension; that is, they valued CE more positively and considered it to be very useful in different facets both at the formative and educational levels and when relating socially, expressing feelings, and self-managing emotionally. These results were contrary to those obtained in past research in which students considered this content to be of little relevance, which could also have been influenced by the teacher’s attitude [29,30]. In this line, in a study by Conesa and Angosto, between 9% and 27% of the teachers of CSE and the Spanish Baccalaureate in Murcia said that they complied with and taught all of the contents related to the block of contents of “artistic and expressive physical activities”; a lack of time and qualifications were given as the main reasons for not complying [31].

Concerning the above, the dimension “Attitude of the teacher” showed the highest scores (M = 3.81); the students were in good agreement with the attitudes shown by their teacher towards the content of CE and its method of transmission. These results were supported by what has been cited in various research studies: student satisfaction and motivation are conditioned by the teacher’s attitude; i.e., the fact that students show positive feelings and interest in a subject is associated with the learning environment generated by the teacher [2,32,33,34]. In this sense, the didactic techniques used by the teacher seem to be influential in the student’s motivation to learn the content [35,36]. Furthermore, in the case of CE content such as dance, a study by Amado et al. found that employing a methodology that alternated direct instruction based on reference models for the students with creative inquiry and involving cognitive participation of the students could increase the motivation of the class, involve boys more cognitively, and satisfy the perception of competence of the girls towards the CE content [37].

For the dimension “Preference”, the lowest scores were obtained (M = 3.11), and students were indifferent to the items related to this dimension. It can be said that this content was not considered very preferential by the students in comparison with other content or other activities carried out in the subject of PE (especially for the male gender). Concerning this, other studies showed how this preference for CE content could be influenced by the expressive and cooperative nature of the content and that boys felt less competent than girls [2,38]. This sensation of lower competence in CE could generate rejection and disinterest, which resulted in children preferring more competitive activities in which they felt more competent [39]. In contrast, girls were more interested in and preferred this content to others due to their high level of competition in it [40].

Finally, in the “liking” dimension, the scores obtained were not very high (M = 3.39): boys (M = 3.19) showed more indifference, and girls (M = 3.58) showed a greater agreement with this dimension. In this sense, it seemed that girls showed a greater interest in CE than boys, and it could be that CE offered girls content that was more closely related to their tastes and motivations. As suggested in other studies, this could be because boys are more motivated by more active, aggressive, or competitive activities that involve greater resistance and strength [19,23,41]. However, girls are more interested in rhythm, coordination, aesthetics, flexibility, and ultimately expressive activities [42,43].

### 4.1. Practical Applications

The results obtained showed a significant difference between genders in the attitudes and perceptions that students had regarding the different dimensions that encompassed the contents of CE. Such content is of great interest in PE because it represents an artistic and expressive element of physical activity that is aimed at promoting personal development, learning values, and improving social and communication skills [44,45]. Therefore, it seems necessary to address these differences between genders regarding the interest and motivation with respect to the content of CE by seeking the assimilation and learning of the knowledge that encompasses this content by all students so that there is a transfer of the content to their daily lives in relation to a better knowledge of the characteristics of the body itself and an adequate control and use of emotions [45,46].

It would be interesting for the educational administrations to advocate for ongoing teacher training courses to instruct teachers on the most appropriate methodologies to work on the diversity of contents that Physical Education comprises in this case. Regarding CE content, this lower positive attitude on the part of boys towards this content could be due to a lower sense of competence in it, thereby generating in boys a greater rejection of CE and demotivation regarding it than in girls [38,39,40].

The use of hybrid methodologies that alternate between direct instruction and creative inquiry could be a solution to develop this learning competence through CE by taking into account the different interests of the student according to gender in which boys become more involved at a cognitive level with inquiry and girls reaffirm their sense of competence towards CE with direct instruction [37].

Teachers should design and employ specific motivational teaching strategies adapted to the CE content in an attempt to create a more task-oriented climate that fosters more positive experiences during practice to favor student development and the assimilation of more volitional behaviors [2]. Using innovative active methodologies such as gamification could help teachers to increase intrinsic motivation, autonomy development, and the development of social skills in their students [47].

### 4.2. Limitations

This was a cross-sectional study; therefore, it was not possible to establish cause–effect relationships. In this sense, in future research it would be interesting to continue exploring these findings to establish causal relationships. 

The sample of the present investigation was limited to students from the Spanish autonomous community of Extremadura, and sociocultural variables may have influenced the results obtained. As a result of this, it would be enriching to develop this type of study in the other autonomous communities of Spain in order to be able to compare the perceptions that students have of CE in different Spanish regions.

Non-binary gender was not taken into account—only the male and female genders were considered.

The method used to collect the responses was an online form; however, as was observed in several previous studies, when compared to a face-to-face interview, respondents may have had difficulties when filling out the questionnaire due to a lack of understanding of some questions that conditioned their responses and caused them to opt for an intermediate answer or to choose not to answer the question [48,49].

## 5. Conclusions

The present research found that girls showed a more positive perception of the CE contents in the PE subject than boys; the latter showed a greater indifference and a lower preference for this content compared to other contents of the subject. However, at a general level, they valued CE with certain positivity in its usefulness at a formative and educational level and in terms of expressing feelings and emotional self-management. Furthermore, concerning the teacher’s attitude towards the content, the students agreed with the methods and means used to transmit the learning of CE.

## Figures and Tables

**Table 1:** Characterization of the sample (N = 889).

Variable	Categories	N	%
Gender	Men	423	47.6
	Women	466	52.4
Age group	12–14 years	415	46.7
	15–17 years	470	52.9
		M	SD
Age		14.58	1.47
Height		1.64	0.01
Weight		56.44	11.97
BMI		20.63	3.46

N: number; %: percentage; M: mean; SD: standard deviation; BMI: body mass index.

**Table 2:** Descriptive data and differences in each of the items of the questionnaire according to gender.

	Gender		
Item	Women	Men	
	M (SD)	M (SD)	*p*
1. Corporal Expression is useful for my training.	3.95 (1.01)	3.69 (1.13)	0.001 **
2. Corporal language allows me to express my feelings.	4.13 (1.02)	3.69 (1.14)	<0.001 **
3. The learning I receive in Corporal Expression is necessary and important.	3.97 (0.98)	3.58 (1.05)	<0.001 **
4. The Corporal Language classes improve my mood.	3.59 (1.09)	3.29 (1.10)	<0.001 **
5. Corporal Language helps me to get to know myself better, relate to others and be creative.	3.77 (1.10)	3.42 (1.13)	<0.001 **
6. Corporal Language contributes to my overall education.	3.50 (1.07)	3.33 (1.15)	0.020
7. Corporal language is good for socializing.	4 (1.01)	3.58 (1.16)	<0.001 **
8. Corporal Expression is a social experience and gives you opportunities to get to know your peers in a deeper way.	3.92 (1.02)	3.57 (1.10)	<0.001 **
9. Corporal Expression classes create a very positive environment.	4.03 (0.96)	3.69 (1.05)	<0.001 **
10. Corporal Expression provides me with significant relief from accumulated stress.	3.74 (1.17)	3.31 (1.15)	<0.001 **
11. Corporal Language also improves your overall health and not just fitness activities.	3.94 (0.98)	3.55 (1.07)	<0.001 **
12. The activities I learn in Corporal Language Arts seem important to me.	3.81 (0.99)	3.45 (1.08)	<0.001 **
13. What I learn in Corporal Expression is of no use to me.	4.23 (1.03)	3.83 (1.18)	<0.001 **
14. I like Corporal Expression because it works on aesthetics and social relations.	3.59 (1.02)	3.42 (1.07)	0.016
15. I don’t like Corporal Language Arts because it doesn’t have as many risks and challenges as sports.	3.75 (1.19)	3.31 (1.21)	0.903
16. I find Corporal Expression interesting because it is not competitive.	3.55 (1.17)	3.06 (1.17)	<0.001 **
17. I prefer Corporal Expression to other content such as sports, physical fitness, etc.	3 (1.25)	2.61 (1.30)	<0.001 **
18. I prefer Corporal Expression because I interact with my classmates more than when we do other Physical Education content.	3.25 (1.19)	2.93 (1.13)	<0.001 **
19. Corporal Language is not as much fun as other content.	3.56 (1.26)	2.87 (1.30)	<0.001 **
20. Corporal Language is more important than the rest of the content of Physical Education.	2.61 (1.05)	2.53 (1.15)	0.159
21. If doing Corporal Expression in Physical Education classes were optional, I would choose to do it.	3.55 (1.18)	2.86 (1.20)	<0.001 **
22. I like Corporal Language Arts because it is something different from what we normally do.	3.74 (1.11)	3.22 (1.13)	<0.001 **
23. I like Corporal Language Arts because it is cooperative.	3.66 (1.06)	3.37 (1.15)	<0.001 **
24. I like the time I spend doing Corporal Language Arts activities.	3.62 (1.12)	3.18 (1.17)	<0.001 **
25. I like Corporal Language Arts because it involves artistic activities.	3.62 (1.08)	3.09 (1.13)	<0.001 **
26. I like Corporal Language Arts because we do more games in it.	3.58 (1.03)	3.34 (1.17)	0.002
27. When I finish the Corporal Language class in Physical Education, I am left wanting more.	3.27 (1.23)	2.96 (1.19)	<0.001 **
28. The teacher values Corporal Language Arts.	3.86 (1.09)	3.72 (1.06)	0.029
29. The teacher offers opportunities for the development of expressive skills.	3.70 (1.10)	3.58 (1.04)	0.041
30. The teacher tries to make the Corporal Expression sessions fun.	4.09 (0.99)	3.95 (1.07)	0.059
31. My P.E. teacher makes the Corporal Expression class useful to me.	3.90 (1.09)	3.69 (1.10)	0.002 **
32. I feel that my P.E. teacher makes learning in Corporal Language valuable to me.	3.88 (1.13)	3.72 (1.09)	0.009

Mann–Whitney U test *p*-values: ** *p* < 0.0016 was significant. M = mean value; SD = standard deviation. Each score obtained was based on a Likert scale (1–5): 1—“Strongly disagree”, 2—“Strongly disagree”, 3—“Indifferent”, 4—“Strongly agree”, and 5—“Strongly agree”.

**Table 3:** Descriptive analysis and differences for each dimension of the instrument.

		Gender			Age Group		
Dimensions	Total	Men	Women	*p*	12–14 Years	15–17 Years	*p*
	M (SD)	M (SD)	M (SD)		M (SD)	M (SD)	
1. Assessment of Corporal Expression	3.70 (0.74)	3.52 (0.75)	3.86 (0.71)	<0.001 **	3.66 (0.75)	3.74 (0.74)	0.191
2. Preference	3.11 (0.74)	2.88 (0.70)	3.32 (0.71)	<0.001 **	3.07 (0.73)	3.15 (0.75)	0.183
3. Pleasure or liking	3.39 (0.90)	3.19 (0.92)	3.58 (0.85)	<0.001 **	3.37 (0.92)	3.42 (0.90)	0.518
4. Teacher’s attitude	3.81 (0.85)	3.73 (0.80)	3.88 (0.89)	0.001 **	3.83 (0.84)	3.80 (0.87)	0.540

Note: ** *p* < 0.016 was significant. M = mean value; SD = standard deviation. Each score obtained was based on a Likert scale (1–5): 1—“Strongly disagree”, 2—“Strongly disagree”, 3—“Indifferent”, 4—“Strongly agree”, 5—“Strongly agree”.

**Table 4:** Correlations between the dimensions and the age group variable.

Dimensions	Age *ρ (p)*	IMC *ρ (p)*
1. Assessment of Corporal Expression	0.063 (0.062)	−0.018 (0.591)
2. Preference	0.060 (0.076)	−0.013 (0.694)
3. Pleasure or liking	0.040 (0.238)	−0.029 (0.381)
4. Teacher’s attitude	−0.002 (0.944)	−0.048 (0.151)

Each score obtained was based on a Likert scale (1–5).

## Data Availability

The datasets used during the current study are available from the corresponding author upon reasonable request.

## References

[1] Bonet M., Menescardi Royuela C. (2022). Análisis de La Actitud Del Alumnado y El Profesorado Ante El Contenido de Expresión Corporal y Los Estereotipos de Género: Resultados de La Experiencia Tras La Realización de Una Unidad Didáctica. Retos Nuevas Tend. En Educ. Física Deporte Recreación.

[2] Sevil J., Abós Á., Aibar A., Julián J.A., García-González L. (2016). Gender and Corporal Expression Activity in Physical Education: Effect of an Intervention on Students’ Motivational Processes. Eur. Phy. Educ. Rev..

[3] Monzó M.P., Martínez S.G., Jaén M.G., Valero A.F. (2021). Goal Orientations and Basic Psychological Needs in the Development of Corporal Expression in Primary Education: A Pilot Study. Retos.

[4] de Andrés Rubio M.N. (1993). La Expresión Corporal En El Segundo Ciclo de Educación Infantil.

[5] Herranz Aragoneses A., López Pastor V.M. (2015). La Expresión Corporal En Educación Infantil. Peonza Rev. Educ. Física Para Paz.

[6] Ramírez L.V., Vidaci A. (2021). Motivation towards the Body Expression Classes. Int. J. Soc. Policy Educ..

[7] Gobierno de España (2022). Real Decreto 157/2022, de 1 de Marzo, Por El Que Se Establecen La Ordenación y Las Enseñanzas Mínimas de La Educación Primaria.

[8] (2022). Gobierno de España Real Decreto 217/2022, de 29 de Marzo, Por El Que Se Establece La Ordenación y Las Enseñanzas Mínimas de La Educación Secundaria Obligatoria.

[9] Boletín Oficial del Estado (2020). Ley Orgánica 3/2020, de 29 de Diciembre, Por La Que Se Modifica La Ley Orgánica 2/2006, de 3 de Mayo, de Educación.

[10] (2022). Diario Oficial de Extremadura DECRETO 110/2022.

[11] Llopis Garrido A., García Montes M.E. (2012). Danza: Experiencias Prácticas de Los Estudiantes de La Diplomatura de Educación Física y de La Licenciatura de Ciencias de La Actividad Física y El Deporte. Tándem Didáctica La Educ. Física.

[12] Moreno J.A., Hellín M.G. (2007). El Interés Del Alumnado de Educación Secundaria Obligatoria Hacia La Educación Física. Rev. Electrónica Investig. Educ..

[13] Archilla Prat M.T., Pérez Brunicardi D. (2012). Dificultades Del Profesorado de E. F. Con Las Actividades de Expresión Corporal En Secundaria. EmásF Rev. Digit. Educ. Física.

[14] Arias García J.R., Fernández Díez B., San Emeterio García C. (2020). Construcción y Validación de Un Instrumento Para La Medida de Las Actitudes Hacia La Expresión Corporal (Construction and Validation of an Instrument for Measuring Attitudes towards Body Expression). Retos.

[15] Robles Rodríguez J., Abad Robles M., Castillo Viera E., Giménez Fuentes-Guerra F., Robles Rodríguez A. (2013). Factores Que Condicionan La Presencia de La Expresión Corporal En La Enseñanza Secundaria Según El Profesorado de Educación Física. RETOS Nuevas Tend. Educ. Física Deporte Educ..

[16] Bortoleto M.A.C., Ontañón Barragán T., Cardani L.T., Funk A., Melo C.C., Santos Rodrigues G. (2020). Gender Participation and Preference: A Multiple-Case Study on Teaching Circus at PE in Brazilians Schools. Front. Educ..

[17] Moya-Mata I., Ros Ros C., Bastida Torróntegui A., Menescardi Royuela C. (2013). Estereotipos de Sexo y Raza En Las Imágenes de Los Libros de Texto de Educación Física En Primaria (Sex and Race Stereotypes in Images of Textbooks in Primary Physical Education). Retos.

[18] Colás Bravo P., Moreno Villaciervos P. (2007). La Interiorización de Los Estereotipos de Género En Jóvenes y Adolescentes. Rev. Investig. Educ..

[19] Pastor-Vicedo J.C., Sánchez-Oliva A., Sánchez-Blanchart J., Martínez-Martínez J. (2019). Estereotipos de Género En Educación Física. Rev. EuroAmericana Cienc. Deporte.

[20] Koca C., Hiilya Af F., Demirhan G. (2005). Attitudes toward Physical Education and Class Preferences of Turkish Adolescents in Terms of School Gender Composition. Adolescence.

[21] Colley A., Comber C., Hargreaves D.J. (1994). Gender Effects in School Subject Preferences: A Research Note. Educ. Stud..

[22] Torrents C., Mateu M., Planas A., Dinusôva M. (2011). Posibilidades de Las Tareas de Expresión Corporal Para Suscitar Emociones En El Alumnado. Rev. Psicol. Deporte.

[23] Ángel J.B., Fernández García E., Sierra Zamorano M.Á. (2007). Estereotipos de Género, Actividad Física y Escuela: La Perspectiva Del Alumnado * Gender Stereotypes, Physical Activity and School: The Perspective of the Students. Profesorado. Rev. Currívulum Form. Profr..

[24] Sicilia Camacho Á., Sáenz-López Buñuel P., Manzano Moreno J.I., Delgado Noguera M.Á. (2009). El Desarrollo Curricular de La Educación Física En Primaria y Secundaria: Un Análisis Desde La Perspectiva Del Profesorado. Apunt. Educ. Física Deportes.

[25] Salkind N.J., Escalona R.L., Valdés Salmerón V. (1999). Métodos de Investigación.

[26] Nunnally J.C., Bernstein I.H. (1994). Psychometric Theory.

[27] Arenas D., Vidal-Conti J., Muntaner-Mas A. (2022). Estereotipos de Género y Tratamiento Diferenciado Entre Chicos y Chicas En La Asignatura de Educación Física: Una Revisión Narrativa (Gender Stereotypes and Differential Treatment between Boys and Girls in Physical Education Subject: A Narrative Review). Retos.

[28] Díaz K.D.C.M., González R.I.S., Gutiérrez M.D.L.Á.G., Durán E.R.v. (2017). Rompiendo Con Los Estereotipos: Una Experiencia Educativa Con Enfoque de Género En Una Escuela Básica. REXE-Rev. Estud. Exp. Educ..

[29] Quevedo-Blasco V.J., Quevedo-Blasco R., Bermúdez M.P. (2009). Análisis de La Motivación En La Práctica de Actividad Físico-Deportiva En Adolescentes. Rev. Investig. Educ..

[30] José J., Martínez A., Nicolás López J., Molina M., Martínez López J. (2015). Preferencias Del Alumnado En Los Contenidos de Educación Física En 3^o^ y 4^o^ de e.S.O. EmásF Rev. Digit. Educ. Física.

[31] Conesa E., Angosto S. (2017). Body Expression and Dance in Physical Education of Secondary and High School. Cuad. Psicol. Deporte.

[32] Zeng H.Z., Hipscher M., Leung R.W. (2011). Attitudes of High School Students toward Physical Education and Their Sport Activity Preferences. J. Soc. Sci..

[33] Subramaniam P.R., Silverman S. (2002). Using Complimentary Data: An Investigation of Student Attitudes in Physical Education. J. Sport Pedagog..

[34] Bores-García D., Hortigüela-Alcalá D., Hernando-Garijo A., González-Calvo G. (2021). Analysis of Student Motivation towards Body Expression through the Use of Formative and Share Assessment. Retos.

[35] Tessier D., Sarrazin P., Ntoumanis N. (2010). The Effect of an Intervention to Improve Newly Qualified Teachers’ Interpersonal Style, Students Motivation and Psychological Need Satisfaction in Sport-Based Physical Education. Contemp. Educ. Psychol..

[36] Ntoumanis N., Barkoukis V., Thøgersen-Ntoumani C. (2009). Developmental Trajectories of Motivation in Physical Education: Course, Demographic Differences, and Antecedents. J. Educ. Psychol..

[37] Amado D., del Villar F., Sánchez-Miguel P.A., Leo F.M., García-Calvo T. (2016). Analysis of the Impact of Creative Technique on the Motivation of Physical Education Students in Dance Content: Gender Differences. J. Create. Behav..

[38] Murillo B., Julián J.A., García-González L., Abarca-Sos A., Zaragoza J. (2014). Influencia Del Género y de Los Contenidos Sobre La Actividad Física y La Percepción de Competencia En Educación Física. RICYDE. Rev. Int. Cienc. Deporte.

[39] O’Neill J.R., Pate R.R., Liese A.D. (2011). Descriptive Epidemiology of Dance Participation in Adolescents. Res. Q Exerc. Sport.

[40] Ruiz L.M., Graupera J.L., Moreno J.A., Rico I. (2010). Social Preferences for Learning among Adolescents in Secondary Physical Education. J. Teach. Phys. Educ..

[41] Canales-Lacruz I., Rey-Cao A. (2014). Student’s Perceptions of Gender Differences with Regards to the Visual and Tactile Interaction Involved in Coporal Expression Activities. Movimento.

[42] Cardoso F.L., Silveira R.A., Zequinão M.A., Martins C., Souza C.A. (2010). Corporal Self-Perception and Motor Preferences of Dance Practitioners. Movimento.

[43] Klomsten A.T., Marsh H.W., Skaalvik E.M. (2005). Adolescents’ Perceptions of Masculine and Feminine Values in Sport and Physical Education: A Study of Gender Differences. Sex Roles.

[44] Redondo-Garrido M.A., Gómez-Carmona C.D., Bastida-Castillo A., Mancha-Triguero D., Gamonales-Puerto J.M. (2019). Are There Differences in the Emotions Perceived by Secondary Education Students Because of Sex and Academic Year in Body Expression Sessions?. Sport Health Phys. Act..

[45] Vidaci A., Vega-Ramírez L., Cortell-Tormo J.M., Karwowski M., Jankowska D.M., Tchounwou P.B. (2021). Development of Creative Intelligence in Physical Education and Sports Science Students through Body Expression. Int. J. Environ. Res. Public Health.

[46] ARRIAgAdA K.R. (2013). El Despertar de Las Emociones. Un Trabajo Corporal. Multiárea.

[47] Pérez M.R., Oliva D.S., Moledo C.P. (2021). Proyecto África “La Leyenda de Faro”: Efectos de Una Metodología Basada En La Gamificación Sobre La Motivación Situacional Respecto al Contenido de Expresión Corporal En Educación Secundaria (Africa Project “La Leyenda de Faro”: Effects of a Methodology. Retos.

[48] Heerwegh D. (2009). Mode Differences Between Face-to-Face and Web Surveys: An Experimental Investigation of Data Quality and Social Desirability Effects. Int. J. Public Opin. Res..

[49] Heerwegh D., Loosveldt G. (2008). Face-to-Face versus Web Surveying in a High-Internet-Coverage PopulationDifferences in Response Quality. Public Opin. Q.

